# Depressive Symptoms Mediate the Association Between Family Functioning and Non-Suicidal Self-Injury in Adolescent Psychiatric Patients: A Cross-Sectional Study

**DOI:** 10.62641/aep.v54i4.2208

**Published:** 2026-08-15

**Authors:** Yushan Zhang, Li Luo, Rui Tang, Xiaoqing Luo, Qi Liu

**Affiliations:** ^1^Mental Health Center, West China Hospital, Sichuan University, 610041 Chengdu, Sichuan, China

**Keywords:** non-suicidal self-injury, family functioning, depressive symptoms, mediation analysis, adolescent psychiatry, emotion regulation

## Abstract

**Background:**

Non-suicidal self-injury (NSSI) is highly prevalent among adolescent psychiatric patients, yet the pathways linking family functioning to NSSI have not been fully elucidated. This study aimed to investigate whether depressive symptoms function as a mediator in the association between family functioning and NSSI among adolescent psychiatric inpatients.

**Methods:**

A cross-sectional study was conducted among 180 adolescent patients admitted to a psychiatric department between June 2023 and June 2025. Family functioning, depressive symptoms, and NSSI-related frequency (encompassing both NSSI ideation and actual NSSI behaviours) were assessed using validated Chinese versions of the Family Adaptability and Cohesion Evaluation Scale II, Patient Health Questionnaire-9 (PHQ-9), and Ottawa Self-Injury Inventory. To avoid measurement overlap between the mediator and outcome, the PHQ-8 total score (PHQ-9 excluding the self-harm/suicide item) was employed as the mediator in the primary analysis. Mediation analysis was performed using the PROCESS macro with bootstrap methods, controlling for demographic and clinical covariates including age, sex, only-child status, parental marital status, family monthly income, duration of illness, history of suicide attempts, and primary diagnosis.

**Results:**

NSSI was identified in 60.56% of participants. Family functioning was negatively correlated with depressive symptoms (r = –0.463, *p* < 0.001) and NSSI-related frequency score (r = –0.387, *p* < 0.001). Depressive symptoms partially mediated the association between family functioning and NSSI-related frequency (indirect effect = −0.058, 95% CI [−0.092, −0.029], accounting for 34.52% of the total effect. Sensitivity analyses including comparison with the original PHQ-9 as mediator (proportion mediated = 40.48%) and a most conservative model utilising only NSSI behavioural items as the outcome (proportion mediated = 35.16%) confirmed the robustness of these findings.

**Conclusions:**

Among adolescents with mental disorders, depressive symptoms may partially mediate the negative association between family functioning and non-suicidal self-injury. Interventions may benefit from integrating family-based approaches with depression-targeted therapies to address NSSI in this vulnerable population.

## Introduction

Non-suicidal self-injury (NSSI) is characterised by intentional, self-directed tissue damage undertaken without suicidal intent and outside socially sanctioned practices [1]. Typical manifestations encompass cutting, burning, scratching, and self-hitting [2]. This behaviour has emerged as a pressing public health concern globally, particularly during adolescence. According to a recent pooled analysis, approximately 22.1% of adolescents worldwide have engaged in NSSI at some point in their lives [3], with this prevalence rising to 24.7% in Chinese youth populations [4]. Notably, prevalence rates are substantially elevated in clinical cohorts, with studies indicating that 40%–60% of adolescent psychiatric patients engage in NSSI [5]. Beyond the immediate physical harm, NSSI confers remarkably downstream risks, including emotional distress, impaired psychosocial functioning, and heightened probability of future suicidal behaviour [6]. In light of these potentially serious consequences, the identification of modifiable determinants and mechanistic pathways of NSSI is essential to inform the development of targeted preventive and therapeutic strategies.

Family functioning, defined as the quality of emotional bonding and adaptability within the family system, has been consistently recognised as a key determinant shaping psychological well-being and behavioural adjustment during adolescence [7]. Family systems theory posits that dysfunctional environments—characterised by low cohesion, poor communication, and inadequate adaptability—may impair adolescents’ emotional regulation and coping capacities, thereby increasing susceptibility to maladaptive behaviours such as NSSI [8, 9]. This theoretical proposition is corroborated by empirical findings. For instance, a large-scale path analysis conducted among Chinese youth revealed a statistically significant positive association between compromised family functioning and NSSI [10]. Similarly, a longitudinal study confirmed that family dysfunction prospectively predicted NSSI urges among adolescents [11].

Despite the well-documented association between family functioning and NSSI, the intervening mechanisms accounting for this association have not been fully elucidated. Depressive symptomatology constitutes a plausible candidate that may partly explain the association between family dysfunction and NSSI. From one perspective, the family environment is considerably associated with the onset and progression of depression, with adverse household climates reliably predicting elevated depressive symptoms in adolescents [12]. From another perspective, depression itself ranks among the strongest individual-level predictors of self-injurious behaviour, as converging evidence indicates that youth experiencing depressive symptoms are substantially more prone to NSSI [13]. Early work by Baetens and colleagues offered initial support for the hypothesis that depressive symptoms operate as an intermediary in the pathway from family dysfunction to NSSI among young people [14].

However, evidence for this mediating pathway derives primarily from community samples, with limited data from clinical psychiatric populations, particularly in China where family dynamics may differ owing to cultural factors. Given the regional diversity in socioeconomic conditions and healthcare resources across China, further validation in different clinical settings is warranted. Additionally, it remains unclear whether this indirect pathway functions uniformly across diverse psychiatric diagnostic categories, such as depressive disorders, bipolar spectrum conditions, and anxiety disorders. Accordingly, this study aimed to determine whether depressive symptoms serves as an intermediary mechanism connecting family functioning to NSSI in adolescents receiving psychiatric care, based on cross-sectional clinical assessment data. The findings may provide further empirical support for integrating family-based and depression-targeted approaches in the prevention and treatment of NSSI in clinical settings.

## Materials and Methods

### Study Design and Participants

This cross-sectional investigation was designed and reported in accordance with the Strengthening the Reporting of Observational Studies in Epidemiology (STROBE) guidelines [15]. All procedures were conducted in conformity with the ethical standards articulated in the Declaration of Helsinki [16]. Ethical approval was obtained from the Ethics Committee of West China Hospital, Sichuan University (approval No. 2023-1517). As this study exclusively analysed pre-existing clinical assessment data collected during standard care and entailed no additional interventions, the Ethics Committee granted a waiver of individual informed consent. All identifying patient information was removed prior to data extraction and subsequent analysis.

Adolescent patients admitted to the psychiatric inpatient unit of West China Hospital, Sichuan University, a tertiary academic medical centre located in an urban area of southwestern China, between June 2023 and June 2025 were consecutively enrolled. Consecutive enrolment was defined as the inclusion of all eligible inpatients who completed routine clinical assessments during the study period, with no selective sampling applied. Participants were included if they: (1) were aged 12–18 years; (2) were diagnosed with a mental disorder according to the International Classification of Diseases, 10th Revision (ICD-10) [17]; (3) had completed assessments of family functioning, depressive symptoms, and NSSI at admission; and (4) had complete medical records. Patients were excluded if they: (1) had comorbid intellectual disability diagnosed according to ICD-10 [17]; (2) were diagnosed with organic mental disorders; (3) were experiencing acute psychotic episodes that precluded reliable completion of self-report measures; (4) had severe physical illnesses that interfered with assessment; or (5) had any missing items on the assessment scales.

**Fig. 1.**
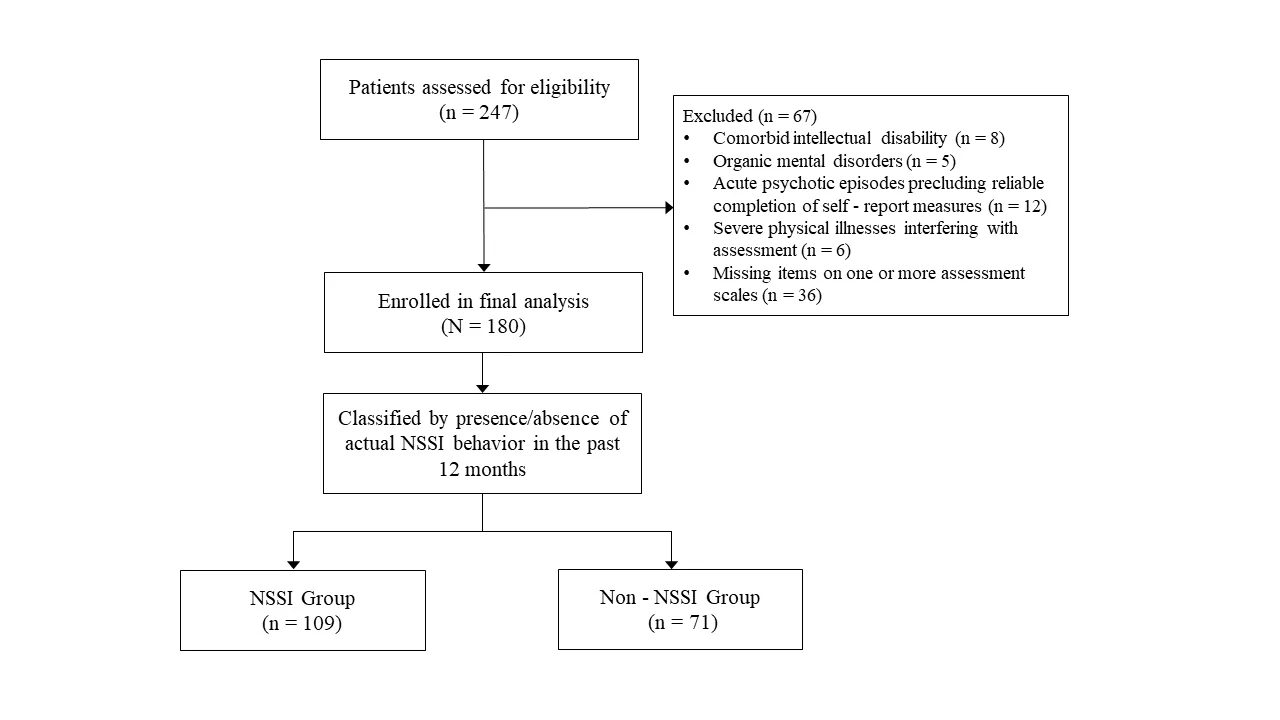
**Flowchart of participant selection**. NSSI, non-suicidal self-injury.

Between June 2023 and June 2025, a total of 247 adolescent inpatients at West China Hospital, Sichuan University were assessed for eligibility. Of these, 67 were excluded for the following reasons: comorbid intellectual disability (n = 8), organic mental disorders (n = 5), acute psychotic episodes precluding reliable completion of self-report measures (n = 12), severe physical illnesses interfering with assessment (n = 6), and missing items on one or more assessment scales (n = 36). Ultimately, 180 patients were enrolled in the final analysis (Fig. 1).

### Data Collection Procedures

Two trained investigators independently retrieved clinical information from the electronic medical records system. Extracted information included: (1) demographic characteristics: age, sex, years of education, only-child status, parental marital status, and family monthly income (Chinese Yuan, CNY); (2) clinical information: primary diagnosis, duration of illness, history of NSSI, and history of suicide attempts; and (3) scores from standardised psychological assessments: family functioning, depressive symptoms, and NSSI. A third researcher verified the extracted data for accuracy, and any discrepancies were resolved through discussion.

### Measures

#### Family Functioning

Family functioning was assessed using the Chinese version of the Family Adaptability and Cohesion Evaluation Scale II (FACES II-CV). The original FACES II was developed by Olson *et al*. [18] based on the Circumplex Model of family systems, and the Chinese version was adapted and validated by Phillips *et al*. [19], demonstrating satisfactory psychometric properties in Chinese populations: test–retest reliability coefficients were 0.84 for cohesion and 0.91 for adaptability, with Cronbach’s α ≥ 0.73 for both dimensions. The scale comprises 30 items measuring two dimensions: family cohesion (16 items) and family adaptability (14 items). Each item is rated on a 5-point Likert scale ranging from 1 (“not at all”) to 5 (“always”), with higher scores indicating superior family cohesion and adaptability. The total score, calculated by summing the cohesion and adaptability subscale scores, was utilised as the indicator of overall family functioning in this study. In the present sample, the Cronbach’s α for the FACES II-CV total scale was 0.89.

#### Depressive Symptoms

Depressive symptoms were evaluated using the Patient Health Questionnaire-9 (PHQ-9), a widely employed self-report screening instrument for depression [20]. The PHQ-9 consists of nine items corresponding to the DSM-5 criteria for major depressive disorder, each rated on a 4-point Likert scale from 0 (“not at all”) to 3 (“nearly every day”) over the past two weeks. Total scores range from 0 to 27, with higher scores indicating greater severity of depressive symptoms. The Chinese version of the PHQ-9 has been validated and shown to possess good reliability and validity, with a Cronbach’s α of 0.86 in the general Chinese population [21] and adequate internal consistency (> 0.84) among Chinese adolescents [22]. In the present sample, the Cronbach’s α was 0.87. PHQ-9 Item 9 assesses thoughts of self-harm or suicide (“Thoughts that you would be better off dead, or of hurting yourself in some way”), which conceptually overlaps with the NSSI ideation items in the outcome measure (Ottawa Self-Injury Inventory [OSI; see below]). To mitigate this construct contamination in the mediation model, the primary mediation analysis employed the PHQ-8 total score (Items 1–8, score range: 0–24), which excludes Item 9. The PHQ-8 has been independently validated as a reliable and clinically equivalent measure of depressive symptom severity that avoids confounding with self-harm constructs [23]. The PHQ-9 total score (including Item 9) was retained for descriptive purposes and as a comparator in sensitivity analyses to quantify the degree of inflation attributable to measurement overlap.

#### Non-Suicidal Self-Injury

NSSI was assessed using the Chinese version of the Ottawa Self-Injury Inventory (OSI). The OSI was originally developed by Nixon and colleagues based on research on affect regulation and addictive features of NSSI in adolescents [24]. The Chinese version [25] comprises five sections: (1) frequency of NSSI ideation and behaviours (6 items), (2) body locations of injury (checklist), (3) specific methods of self-injury (checklist), (4) functions of NSSI (15 items across three subscales: social influence, external emotion regulation, and internal emotion regulation, each rated on a 5-point scale from 0 “never” to 4 “always,” scored as the mean of each subscale), and (5) addictive features of NSSI (7 items, each rated 0–4, summed to yield a total score ranging from 0 to 28, with higher scores indicating greater addictive characteristics). The revised scale demonstrated good reliability and validity in Chinese adolescent psychiatric populations, with Cronbach’s α coefficients of 0.799 for NSSI frequency, 0.798 for addictive features, and 0.835 for functional scales [25]. In the present study, the NSSI frequency section was utilised as the primary outcome measure. This section consists of 6 items assessing the occurrence and frequency of both NSSI ideation and actual NSSI behaviours across three progressively longer timeframes: past 1 month (2 items, each scored 0–3), past 6 months (2 items, each scored 0–4), and past 12 months (2 items, each scored 0–4). Within each timeframe, one item assesses NSSI ideation and one assesses actual NSSI behaviours. The total frequency score is calculated by summing all six items (score range: 0–22), with higher scores indicating greater frequency of NSSI. The total frequency score (hereafter termed the “NSSI-related frequency score,” as it encompasses both NSSI ideation and actual NSSI behaviours) was employed as the continuous outcome variable in mediation analyses. For descriptive and group comparison analyses, participants were classified into the NSSI group if they reported any actual NSSI behaviour (e.g., cutting, burning, scratching, or self-hitting) within the past 12 months, and into the non-NSSI group if no actual NSSI behaviour was reported during this period. In the present sample, the Cronbach’s α for the NSSI frequency section was 0.82.

### Statistical Analysis

All statistical procedures were executed using IBM SPSS Statistics version 26.0 (IBM Corp., Armonk, NY, USA). The normality of continuous variables was assessed using the Shapiro–Wilk test. Normally distributed continuous variables were summarised as mean ± standard deviation (Mean ± SD) and compared via independent samples t-tests where appropriate. Non-normally distributed continuous data were reported as median (interquartile range, IQR) and analysed using the Mann–Whitney U test. Categorical data were presented as frequencies (percentages) and evaluated using chi-square tests. A two-tailed significance threshold of *p* < 0.05 was applied throughout.

As all study variables were measured via self-administered instruments, the potential for common method variance was evaluated through Harman’s single-factor test. Specifically, all items across the three scales were entered into an unrotated exploratory factor analysis, and common method bias was deemed negligible if the variance explained by the first extracted factor fell below the 40% benchmark.

Unadjusted bivariate associations among family functioning, depressive symptoms, and NSSI-related frequency score were first explored using zero-order Pearson correlation coefficients. Correlation magnitudes were interpreted as weak (|r| < 0.3), moderate (0.3 ≤ |r| < 0.5), or strong (|r| ≥ 0.5). This preliminary analysis characterised the pairwise associations before covariate-adjusted mediation testing.

The indirect effect of family functioning on NSSI via depressive symptoms was evaluated using Hayes’ PROCESS macro (Model 4) for SPSS [26]. This simple mediation framework decomposes the total effect of the independent variable (X) on the dependent variable (Y, path c) into two components: a direct effect (path c’) and an indirect effect channelled through a single mediator (M), computed as the product of path a (X → M) and path b (M → Y, adjusting for X). Family functioning (FACES II-CV total score) was specified as the independent variable (X), depressive symptoms (PHQ-8 total score, i.e., PHQ-9 excluding Item 9; see Measures) as the proposed mediator (M), and NSSI-related frequency score (OSI frequency score) as the outcome variable (Y). Based on prior evidence and observed baseline group differences, the following covariates were entered into the model: age, sex, only-child status, parental marital status, family monthly income (log-transformed given its skewed distribution), duration of illness, history of suicide attempts, and primary diagnostic category [10, 12]. Statistical significance of the indirect effect was determined using the bias-corrected nonparametric percentile bootstrap approach with 5,000 resamples to construct 95% confidence intervals (CIs), whereby an interval excluding zero constituted a significant mediation effect. The total effect (c), direct effect (c’), and indirect effect (a × b) were reported, with the proportion mediated calculated as |a × b / c| × 100%.

The stability of the primary findings was further evaluated through a series of sensitivity analyses, all of which incorporated the same set of covariates as the main model unless otherwise specified. These supplementary analyses included: (1) re-estimation of the mediation model using a binary NSSI outcome (presence vs. absence) within a logistic regression framework; (2) a subgroup analysis confined to patients with depressive disorders, in which primary diagnostic category was excluded as a covariate given its invariance within this subset; (3) separate mediation models substituting family cohesion and family adaptability as independent variables in place of the composite family functioning score; and (4) re-estimation of the main mediation model excluding clinical covariates (duration of illness, history of suicide attempts, and primary diagnostic category) to assess the potential influence of overadjustment bias, retaining only demographic covariates. To evaluate the effect of measurement overlap between the mediator and outcome, two additional sensitivity analyses were performed: (5) re-estimation using the original PHQ-9 score (including Item 9) as the mediator, to quantify the degree of inflation attributable to the overlap between Item 9 and the NSSI ideation items in the OSI; and (6) re-estimation using PHQ-8 as the mediator with only the three OSI behavioural items (excluding NSSI ideation items) as the outcome variable, representing the most conservative test of the mediation hypothesis with no content overlap between the mediator and outcome.

## Results

### Participant Characteristics

A total of 180 adolescent psychiatric patients were included in this study. The mean age was 15.24 ± 1.76 years (range: 12–18 years), with 112 (62.22%) being female. The majority of participants were diagnosed with depressive disorders (n = 82, 45.56%), followed by bipolar disorders (n = 35, 19.44%), anxiety disorders (n = 31, 17.22%), and other disorders (n = 32, 17.78%). NSSI was identified in 109 (60.56%) participants. Detailed demographic and clinical characteristics are presented in Table 1. Compared with the non-NSSI group, patients in the NSSI group were more likely to be female (χ^2^ = 6.847, *p* = 0.009), had higher rates of parental divorce (χ^2^ = 5.274, *p* = 0.022), longer duration of illness (Z = −2.524, *p* = 0.012), and were more likely to have a history of suicide attempts (χ^2^ = 16.482, *p* < 0.001). Family monthly income showed a trend toward being lower in the NSSI group, though this difference did not attain statistical significance (Z = −1.846, *p* = 0.065). No significant differences were observed between groups in age, only-child status, or primary diagnosis (all *p* > 0.05).

**Table 1.** **Demographic and clinical characteristics of participants (N = 180)**.

Variable		Total (N = 180)	NSSI group (n = 109)	Non-NSSI group (n = 71)	t/χ^2^/Z	*p*
Age (years), Mean ± SD		15.24 ± 1.76	15.18 ± 1.82	15.34 ± 1.67	−0.597	0.551
Sex, n (%)					6.847	0.009
	Male	68 (37.78)	33 (30.28)	35 (49.30)		
	Female	112 (62.22)	76 (69.72)	36 (50.70)		
Only child, n (%)					0.284	0.594
	Yes	95 (52.78)	56 (51.38)	39 (54.93)		
	No	85 (47.22)	53 (48.62)	32 (45.07)		
Parental marital status, n (%)					5.274	0.022
	Intact	132 (73.33)	74 (67.89)	58 (81.69)		
	Divorced/Separated	48 (26.67)	35 (32.11)	13 (18.31)		
Family monthly income (CNY), Median (IQR)		10,000 (6000–15,000)	9,000 (5500–14,000)	11,000 (7000–18,000)	−1.846^a^	0.065
Duration of illness (months), Median (IQR)		12.0 (6.0–20.0)	13.0 (7.0–22.0)	10.0 (6.0–16.0)	−2.524^a^	0.012
History of suicide attempts, n (%)					16.482	<0.001
	Yes	58 (32.22)	48 (44.04)	10 (14.08)		
	No	122 (67.78)	61 (55.96)	61 (85.92)		
Primary diagnosis, n (%)					4.582	0.205
	Depressive disorders	82 (45.56)	54 (49.54)	28 (39.44)		
	Bipolar disorders	35 (19.44)	22 (20.18)	13 (18.31)		
	Anxiety disorders	31 (17.22)	15 (13.76)	16 (22.54)		
	Others	32 (17.78)	18 (16.51)	14 (19.72)		

Note: NSSI, non-suicidal self-injury; SD, standard deviation; IQR, interquartile range; CNY, Chinese Yuan. Continuous variables with normal distribution were compared using independent samples t-tests; continuous variables with skewed distribution were compared using Mann−Whitney U test (^a^ denotes Z value); categorical variables were compared using chi-square tests.

### Descriptive Statistics and Group Comparisons

The mean scores for family functioning (FACES II-CV) and depressive symptoms (PHQ-9) were 60.37 ± 12.85 and 14.28 ± 5.63, respectively. As the primary mediation analysis employed the PHQ-8 (PHQ-9 excluding Item 9), the PHQ-8 total score was also computed: the mean PHQ-8 score was 12.96 ± 5.18 for the total sample (NSSI group: 15.07 ± 4.38; non-NSSI group: 9.72 ± 4.86). The Shapiro−Wilk test indicated that NSSI-related frequency scores (OSI frequency) were significantly non-normally distributed (*p* < 0.001); therefore, OSI scores were reported as median (IQR): 9.0 (3.0–13.0) for the total sample. As shown in Table 2, the NSSI group reported significantly lower FACES II-CV scores (*p* < 0.001), including both family cohesion (*p* < 0.001) and family adaptability (*p* < 0.001). PHQ-9, PHQ-8, and OSI scores were significantly higher in the NSSI group (*p* < 0.001).

**Table 2.** **Comparison of scale scores between NSSI and non-NSSI groups (N = 180)**.

Variable	Total (N = 180)	NSSI group (n = 109)	Non-NSSI group (n = 71)	t/Z	*p*
FACES II-CV, Mean ± SD	60.37 ± 12.85	55.82 ± 11.47	67.36 ± 11.93	−6.129	<0.001
Family cohesion	35.64 ± 8.12	32.78 ± 7.35	40.03 ± 7.56	−6.065	<0.001
Family adaptability	24.73 ± 5.89	23.04 ± 5.42	27.33 ± 5.68	−4.865	<0.001
PHQ-9, Mean ± SD	14.28 ± 5.63	16.54 ± 4.72	10.81 ± 5.28	7.835	<0.001
PHQ-8, Mean ± SD	12.96 ± 5.18	15.07 ± 4.38	9.72 ± 4.86	7.468	<0.001
NSSI-related frequency score (OSI), Median (IQR)	9.0 (3.0-13.0)	13.0(10.0-14.0)	1.0 (0.0-4.0)	−10.876^a^	<0.001

Note: NSSI, non-suicidal self-injury; FACES II-CV, Family Adaptability and Cohesion Evaluation Scale II, Chinese Version; PHQ-9, Patient Health Questionnaire-9; PHQ-8, PHQ-9 excluding Item 9 (self-harm ideation); OSI, Ottawa Self-Injury Inventory; SD, standard deviation; IQR, interquartile range. Normality was assessed using the Shapiro−Wilk test. Continuous variables with normal distribution were compared using independent samples t-tests; ^a^ denotes Z value from Mann−Whitney U test for non-normally distributed variables.

### Common Method Bias Testing

To evaluate the potential influence of common method variance, Harman’s single-factor test was performed. The unrotated exploratory factor analysis yielded 12 factors with eigenvalues exceeding 1.0, and the first extracted factor explained 28.63% of the total variance. As this value fell below the established 40% criterion, common method bias was considered unlikely to have substantially affected the study results.

### Correlations

Table 3 presents the zero-order correlation matrix. Family functioning exhibited significant inverse associations with depressive symptoms (r = −0.463, *p* < 0.001) and NSSI-related frequency score (r = −0.387, *p* < 0.001), while depressive symptoms demonstrated a significant positive association with NSSI-related frequency score (r = 0.524, *p* < 0.001). When PHQ-8 scores (excluding Item 9) were employed in place of PHQ-9, the correlations remained significant and comparable in magnitude: family functioning and PHQ-8 (r = −0.441, *p* < 0.001), PHQ-8 and NSSI-related frequency score (r = 0.498, *p* < 0.001), indicating that the inter-variable associations were not driven by the self-harm ideation item. This pattern of inter-variable associations offered initial evidence consistent with the proposed mediation hypothesis.

**Table 3.** **Zero-order correlation matrix of study variables (N = 180)**.

Variable	1	2	3	4	5
1. Family functioning	—				
2. Family cohesion	0.924***	—			
3. Family adaptability	0.876***	0.673***	—		
4. Depressive symptoms	−0.463***	−0.448***	−0.385***	—	
5. NSSI-related frequency score	−0.387***	−0.372***	−0.318***	0.524***	—

Note: *** *p* < 0.001. NSSI, non-suicidal self-injury; PHQ-9, Patient Health Questionnaire-9 (Items 1–9); PHQ-8, 8-item Patient Health Questionnaire (PHQ-9 excluding Item 9 [self-harm ideation]). PHQ-8 correlations: family functioning and PHQ-8, r = −0.441***; PHQ-8 and NSSI-related frequency score, r = 0.498***.

### Mediation Analysis

**Fig. 2.**
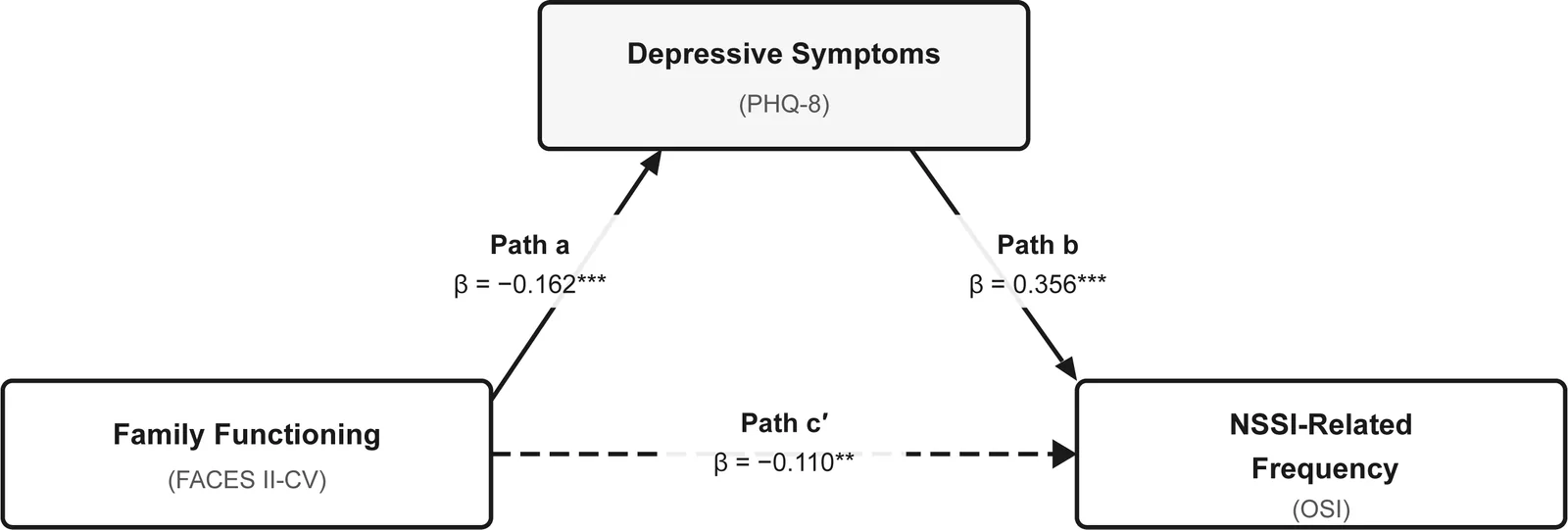
**Mediation model of depressive symptoms on the association between family functioning and NSSI**. The mediation model illustrating the associations among family functioning, depressive symptoms, and NSSI-related frequency in adolescent psychiatric patients (N = 180). Path a represents the effect of family functioning on depressive symptoms; path b represents the effect of depressive symptoms on NSSI-related frequency; path c’ represents the direct effect of family functioning on NSSI-related frequency after controlling for depressive symptoms. The indirect effect (a × b) = −0.058 (Boot SE = 0.016, 95% CI [−0.092, −0.029]), indicating that depressive symptoms partially mediated the association between family functioning and NSSI (proportion of mediation = 34.52%). Unstandardised coefficients are presented. Covariates (age, sex, only-child status, parental marital status, family monthly income, duration of illness, history of suicide attempts, and primary diagnosis) controlled but not shown. NSSI, non-suicidal self-injury; NSSI-related frequency score, OSI frequency section total encompassing both NSSI ideation and actual behaviours; OSI, Ottawa Self-Injury Inventory; PHQ-8, Patient Health Questionnaire-8 (PHQ-9 excluding Item 9); FACES II-CV, Family Adaptability and Cohesion Evaluation Scale II, Chinese Version; Boot SE = bootstrap standard error; CI, confidence interval. ** *p* < 0.01; *** *p* < 0.001.

The results of the primary mediation analysis using the PROCESS macro (Model 4) with the PHQ-8 total score as the mediator are presented in Table 4 and Fig. 2.

**Table 4.** **Mediation effect of depressive symptoms (PHQ-8) on the association between family functioning and NSSI (N = 180)**.

Path		β	SE	t	*p*	95% CI
Direct effects						
	Family functioning → Depressive symptoms (a)	−0.162	0.031	−5.226	<0.001	[−0.223, −0.101]
	Depressive symptoms →NSSI-related frequency score (b)	0.356	0.083	4.289	<0.001	[0.192, 0.520]
	Family functioning →NSSI-related frequency score (c’)	−0.110	0.035	−3.143	0.002	[−0.179, −0.041]
Total and indirect effects						
	Total effect (c)	−0.168	0.035	−4.800	<0.001	[−0.237, −0.099]
	Indirect effect (a × b)	−0.058	0.016	—	—	[−0.092, −0.029]^a^

Note: β, unstandardised regression coefficient; SE, standard error; CI, confidence interval; NSSI, non-suicidal self-injury; PHQ-9, Patient Health Questionnaire-9 (Items 1–9); PHQ-8, 8-item Patient Health Questionnaire (PHQ-9 excluding Item 9 [self-harm ideation]); OSI, Ottawa Self-Injury Inventory. The mediator was the PHQ-8 total score to avoid measurement overlap with the NSSI ideation items in the OSI outcome variable. Covariates included age, sex, only-child status, parental marital status, family monthly income (log-transformed), duration of illness, history of suicide attempts, and primary diagnostic category. ^a^ Bootstrap 95% confidence interval (5000 resamples); the indirect effect is significant as the CI does not include zero. Proportion of mediation = |indirect effect / total effect| × 100% = |−0.058 / −0.168| × 100% = 34.52%.

After controlling for age, sex, only-child status, parental marital status, family monthly income (log-transformed), duration of illness, history of suicide attempts, and primary diagnostic category, family functioning was significantly associated with depressive symptoms (a path: β = −0.162, SE = 0.031, t = −5.226, *p* < 0.001), and depressive symptoms were significantly associated with NSSI-related frequency score (b path: β = 0.356, SE = 0.083, t = 4.289, *p* < 0.001). The total effect of family functioning on NSSI-related frequency score was significant (c path: β = −0.168, SE = 0.035, t = −4.800, *p* < 0.001). When depressive symptoms were included in the model, the direct effect of family functioning on NSSI-related frequency score remained significant but was attenuated (c’ path: β =−0.110, SE = 0.035, t = −3.143, *p* = 0.002).

The indirect effect of family functioning on NSSI-related frequency score through depressive symptoms was statistically significant (a × b = −0.058, Boot SE = 0.016, 95% CI [−0.092, −0.029]). The 95% bootstrap confidence interval did not include zero, confirming the significance of the mediation effect. The proportion of the mediated effect was 34.52%, indicating that depressive symptoms partially mediated the association between family functioning and NSSI.

### Sensitivity Analyses

Sensitivity analyses were conducted to assess the robustness of the findings (Table 5). To evaluate the effect of the measurement overlap identified between PHQ-9 Item 9 (self-harm ideation) and the NSSI ideation items in the OSI, two additional specifications were tested (Table 5, Panel A). When the original PHQ-9 total score (including Item 9) was employed as the mediator (Model A), the indirect effect was −0.068 (Boot SE = 0.018, 95% CI [−0.106, −0.036]; proportion mediated = 40.48%), which was larger than the PHQ-8-based primary estimate (−0.058), suggesting that the inclusion of Item 9 inflated the mediation effect. When the outcome was further restricted to the three OSI behavioural items only—eliminating all content overlap between the mediator and outcome (Model B)—the indirect effect was −0.045 (Boot SE = 0.015, 95% CI [−0.078, −0.019]; proportion mediated = 35.16%). Across the three specifications (primary PHQ-8 model, PHQ-9 model, and PHQ-8 + behavioural OSI model), the indirect effect remained statistically significant, demonstrating that the mediation finding is robust and not a potential construct overlap.

**Table 5.** **Sensitivity analyses for mediation effects**.

Analysis		Mediator	Outcome	Indirect effect	Boot SE	95% CI
Panel A: Measurement overlap analyses						
	Model A: PHQ-9 as mediator (comparison)	PHQ-9	OSI total (6 items)	−0.068	0.018	[−0.106, −0.036]
	Primary model: PHQ-8 as mediator	PHQ-8	OSI total (6 items)	−0.058	0.016	[−0.092, −0.029]
	Model B: PHQ-8 + behavioural OSI only	PHQ-8	OSI behavioural (3 items)	−0.045	0.015	[−0.078, −0.019]
Panel B: Other sensitivity analyses						
	NSSI as dichotomous variable	PHQ-8	NSSI (yes/no)	−0.043	0.017	[−0.080, −0.014]
	Subgroup: Depressive disorders only (n = 82)	PHQ-8	OSI total (6 items)	−0.068	0.026	[−0.124, −0.023]
	Family cohesion as IV	PHQ-8	OSI total (6 items)	−0.053	0.016	[−0.087, −0.025]
	Family adaptability as IV	PHQ-8	OSI total (6 items)	−0.042	0.016	[−0.076, −0.015]
	Excluding clinical covariates^b^	PHQ-8	OSI total (6 items)	−0.063	0.018	[−0.101, −0.031]

Note: All indirect effects represent the path: family functioning (FACES II-CV) → depressive symptoms (mediator) → NSSI-related frequency score (outcome), i.e., a × b, unless the Analysis column specifies a different IV or outcome. IV, independent variable; Boot SE, bootstrap standard error; CI, confidence interval. PHQ-9, Patient Health Questionnaire-9 (Items 1–9); PHQ-8, 8-item Patient Health Questionnaire (PHQ-9 excluding Item 9 [self-harm ideation]). OSI total, Ottawa Self-Injury Inventory frequency score (6 items, including NSSI ideation and behaviours); OSI behavioural, Ottawa Self-Injury Inventory behavioural items only (3 items, excluding NSSI ideation items); NSSI, non-suicidal self-injury; FACES II-CV, Family Adaptability and Cohesion Evaluation Scale II, Chinese Version. All confidence intervals were calculated using 5,000 bootstrap resamples. Panel A presents analyses addressing measurement overlap between the mediator and outcome. Model A retains the original PHQ-9 as the mediator for comparison; the primary model uses PHQ-8; Model B applies the most conservative specification by simultaneously removing Item 9 from the mediator and NSSI ideation items from the outcome. All sensitivity analyses adjusted for the same covariates as the main analysis (age, sex, only-child status, parental marital status, family monthly income, duration of illness, history of suicide attempts, and primary diagnostic category), except as noted. The subgroup analysis of depressive disorder patients excluded primary diagnostic category as a covariate given its invariance within this subset. ^b^The reduced-covariate model adjusted only for demographic covariates (age, sex, only-child status, parental marital status, and family monthly income), excluding duration of illness, history of suicide attempts, and primary diagnostic category to assess potential overadjustment bias. Proportion mediated: primary model = 34.52%; Model A = 40.48%; Model B = 35.16%; reduced-covariate model = 37.50%.

When NSSI was treated as a dichotomous variable, the indirect effect of family functioning on NSSI through depressive symptoms (PHQ-8) remained significant (indirect effect = −0.043, Boot SE = 0.017, 95% CI [−0.080, −0.014]). In the subgroup of patients with depressive disorders (n = 82), the indirect effect was −0.068 (Boot SE = 0.026, 95% CI [−0.124, −0.023]), consistent with the main analysis. When family cohesion and adaptability were analysed separately, both showed significant indirect effects on NSSI through depressive symptoms (family cohesion: indirect effect = −0.053, 95% CI [−0.087, −0.025]; family adaptability: indirect effect = −0.042, 95% CI [−0.076, −0.015]). To address potential overadjustment bias, the model was re-estimated excluding clinical covariates (duration of illness, history of suicide attempts, and primary diagnostic category) that may lie on the causal pathway. The indirect effect remained significant (indirect effect = −0.063, Boot SE = 0.018, 95% CI [−0.101, −0.031]; proportion mediated = 37.50%). Across all sensitivity analyses, the findings were consistent with the main analysis.

Given significant non-normality of the NSSI-related frequency score distribution (Shapiro−Wilk W = 0.934, *p* < 0.001), Spearman correlations were computed as a supplementary check. The results were consistent with Pearson correlations: family functioning was inversely associated with depressive symptoms (ρ = −0.451, *p* < 0.001) and NSSI-related frequency score (ρ = −0.369, *p* < 0.001), and depressive symptoms were positively associated with NSSI-related frequency score (ρ = 0.508, *p* < 0.001).

## Discussion

The current study investigated whether depressive symptoms function as an intermediary mechanism in the association between family functioning and NSSI among adolescents receiving psychiatric treatment. Depressive symptoms partially mediated this association, accounting for 34.52% of the total effect. These observations lend empirical support to the proposed theoretical model whereby impaired family relational dynamics are associated with NSSI through their association with depressive psychopathology, and highlight the potential value of concurrently addressing family-level and individual-level processes in the clinical management of NSSI in adolescent cohorts.

NSSI was reported by 60.56% of adolescent psychiatric patients, consistent with previous research demonstrating elevated NSSI prevalence in clinical cohorts compared with community samples [27]. A recent meta-analysis estimated that approximately 22.1% of adolescents have engaged in NSSI at some point during their lifetime [3], while prevalence in clinical adolescent samples ranges from 40% to 60% [5]. The higher prevalence observed in our sample may reflect the severity of psychopathology and the accumulation of risk factors inherent in clinical populations seeking psychiatric care. Of particular relevance, our results align with a multi-centre investigation by He *et al*. [28], which reported that 43.3% of Chinese adolescents diagnosed with depression had engaged in NSSI, and that family closeness and flexibility served as meaningful buffers against such behaviours.

The significant negative correlation between family functioning and NSSI corroborates existing literature emphasising the critical role of the family environment in adolescent mental health. Wang *et al*. [10] demonstrated in a large-scale structural equation modelling analysis involving 5854 adolescents that poor family functioning was positively associated with NSSI. Our findings extend this evidence by demonstrating a statistical mediation pathway in a clinical sample, where the observed associations may be more pronounced. Family systems theory posits that family cohesion and adaptability serve as fundamental protective factors against emotional and behavioural problems [29]. When these dimensions are compromised, adolescents may experience diminished emotional support, ineffective communication, and heightened exposure to family conflict—all of which are associated with psychological vulnerability.

The mediating role of depressive symptoms can be interpreted within multiple theoretical frameworks. A systematic review indicates that maladaptive family environments may function as chronic psychosocial stressors, progressively eroding adolescents’ psychological resources, and increasing susceptibility to depressive psychopathology [30]. A recent longitudinal network analysis by Zhang *et al*. [31] revealed that family functioning exhibited the strongest bridging effect among risk factors for adolescent psychopathology, with emotional neglect serving as a pivotal node connecting family dysfunction to negative emotional outcomes. Once depressive symptoms emerge, they may precipitate a cascade of negative cognitive and emotional processes linked to heightened risk of NSSI engagement. In this regard, the emotional cascade model advanced by Selby *et al*. [32] offers a theoretically robust account of this process, positing that repetitive focus on aversive affective experiences triggers a progressive amplification of emotional arousal, which may ultimately lead individuals toward NSSI as a dysfunctional strategy for restoring emotional equilibrium.

From the perspective of emotion regulation theory, NSSI predominantly serves to attenuate overwhelming negative affective states, particularly those arising in the context of depressive psychopathology. Consistent with this view, Taylor *et al*. [33] reported that intrapersonal regulatory motives account for 66%–81% of NSSI engagement, with distress relief being the most commonly endorsed function. Adolescents raised in families characterised by low cohesion and poor adaptability may fail to develop adaptive emotion regulation skills, increasing vulnerability to depression and, consequently, NSSI when experiencing emotional distress [12, 34]. Apicella *et al*. [35] emphasised that emotional dysregulation represents a core mechanism underlying NSSI, and this dysregulation often originates from early family experiences that shape the development of self-regulatory capacities.

The finding that depressive symptoms partially mediate the association between family functioning and NSSI suggests that family dysfunction may influence NSSI through additional pathways not examined in this study. Emotion dysregulation represents a particularly strong candidate, as converging evidence indicates that adolescents raised in families with poor cohesion and communication are less likely to develop adaptive emotion regulation skills, thereby increasing their vulnerability to NSSI as a means of managing intolerable affective states [36, 37]. Attachment insecurity constitutes another plausible mechanism; longitudinal evidence links insecure attachment—particularly attachment anxiety—to NSSI onset through diminished self-esteem and impaired cognitive reappraisal capacities [38]. Impulsivity may further contribute to this pathway, as impulsive responding under emotional distress has been consistently associated with NSSI engagement in adolescents [35]. Additionally, adverse childhood experiences such as emotional abuse, neglect, and exposure to family violence have been identified as independent risk factors operating through both emotional and interpersonal pathways [9, 39]. Collectively, these findings suggest that the indirect effect identified in the present study represents one important pathway within a broader multilevel model linking family functioning to NSSI. Future research should employ multiple mediation or sequential mediation designs to simultaneously examine these complementary mechanisms.

These findings have important clinical implications. The partial mediation model supports a multi-level approach to NSSI intervention in adolescent psychiatric patients, addressing both family-level and individual-level factors [4, 40]. At the family level, family-based therapies that enhance cohesion, improve communication, and increase adaptability may mitigate family-level risk factors for NSSI [41]. Bean *et al*. [42] reviewed family therapy for adolescent NSSI and concluded that interventions targeting family communication and cohesion demonstrated efficacy in reducing self-injurious behaviours. At the individual level, treatments targeting depressive symptoms and emotion regulation skills, such as DBT-A, have demonstrated robust short-term abstinence from NSSI (Flygare *et al*., 2025). [43] A recent scoping review by Fang *et al*. [44] found that combining DBT-A with family-system interventions (e.g., Satir therapy) concurrently addresses the emotion-dysregulation pathway and the family-environmental factors underpinning adolescent depression with NSSI, concluding that such integrated biological-behavioural-family strategies are pivotal for managing this comorbidity.

Routine screening for both family functioning and depressive symptoms is essential when evaluating adolescents with NSSI, as impaired family relational quality may be associated with NSSI both directly and indirectly through depressive symptoms. Early identification of these risk factors may facilitate timely intervention before NSSI behaviours become entrenched. Integrating brief family functioning screening tools, such as the FACES II-CV, into routine intake assessments may help identify family-level risk factors. However, implementation in Chinese psychiatric settings may face challenges including mental health stigma, limited caregiver availability, and uneven distribution of mental health resources across regions, all of which warrant further investigation.

Several methodological limitations warrant consideration. The cross-sectional design precludes causal inference regarding the directionality and temporal ordering of the observed associations. Alternative causal sequences cannot be excluded: adolescent NSSI may disrupt family functioning, and depression may emerge as a consequence rather than a precursor of self-injury. Prospective designs are needed to disentangle these temporal relationships. Although we adjusted for multiple potential confounders (age, sex, only-child status, parental marital status, family monthly income, duration of illness, history of suicide attempts, and primary diagnostic category), residual confounding remains possible. This study relied on data extracted from existing medical records, in which important variables such as adverse childhood experiences, substance use, and dietary patterns were not routinely documented, consequently, these potential confounders could not be included, which may have inflated the estimated mediation effect. Future prospective studies should incorporate comprehensive assessment of these potential confounders. Only depressive symptoms were examined as a mediator in this study. The absence of other plausible mechanisms limit the comprehensiveness of the mediation model, and future studies employing multiple mediation or sequential mediation designs are warranted. Exclusive use of self-administered questionnaires introduces the potential for recall inaccuracy and socially desirable responding, although Harman’s single-factor test indicated that common method variance did not substantially threaten data integrity. Furthermore, the primary outcome measure (OSI frequency section) captures both NSSI ideation and actual NSSI behaviours within a single composite score; consequently, the NSSI-related frequency score may not fully distinguish between ideation and action. When the original PHQ-9 (including Item 9) was employed as the mediator, the proportion mediated was 40.48%, compared with 34.52% in the primary PHQ-8 model, suggesting that approximately 6 percentage points were attributable to content overlap rather than a substantive psychological mechanism. The most conservative model, utilising PHQ-8 as the mediator and only OSI behavioural items as the outcome, yielded a proportion mediated of 35.16%, confirming that the mediation pathway is not an artifact of measurement overlap.

Some covariates controlled in the main analysis, such as primary diagnosis, history of suicide attempts, and illness duration, may lie downstream of depressive symptoms rather than function as true confounders, potentially introducing overadjustment bias that attenuates the estimated indirect effect [45]. However, sensitivity analyses excluding these covariates yielded consistent results, suggesting that overadjustment did not substantially alter our conclusions. Additionally, data were collected from a single institution, caution is therefore warranted when extrapolating these findings to other clinical environments or sociocultural settings. Chinese family structures and attitudes toward mental health may differ from those in Western countries, potentially influencing the strength and nature of the observed relationships. Although the sample size was sufficient to identify medium-magnitude effects within the mediation framework, statistical power may have been inadequate for detecting smaller effects or testing more elaborate conditional process models. Subsequent investigations utilising larger, geographically diverse cohorts and prospective methodologies are encouraged. Incorporating objective measures of family functioning, such as observational coding of family interactions, could strengthen assessment validity. Future studies should systematically collect data on adverse childhood experiences, substance use, and other behavioural factors to enable more comprehensive adjustment for potential confounders in mediation models.

## Conclusions

This study provides evidence for empirical support for a statistical mediation model in which depressive symptoms partially mediated the association between family functioning and NSSI. This mediation effect was robust across multiple analytic strategies designed to address measurement overlap between the mediator and outcome, with the most conservative estimate (PHQ-8 as mediator, OSI behavioural items only as outcome) confirming a significant indirect pathway. These findings underscore the importance of considering both family environmental factors and individual psychological processes in understanding and treating NSSI. Clinicians working with this vulnerable population may consider implement comprehensive assessments that evaluate family dynamics alongside depressive symptomatology, and treatment approaches may benefit from integrating family-based interventions with evidence-based therapies targeting emotion regulation and depression.

## Availability of Data and Materials

The data are not publicly available due to ethical restrictions and patient privacy protection. The data presented in this study are available on request from the corresponding author.
